# Visual and quantitative perfusion analysis in left main stem disease: a CE-MARC substudy

**DOI:** 10.1186/1532-429X-15-S1-P195

**Published:** 2013-01-30

**Authors:** John P Greenwood, Ananth Kidambi, Neil Maredia, Kevin Mohee, Steven Sourbron, Manish Motwani, Akhlaque Uddin, David P Ripley, Bernhard A Herzog, Arshad Zaman, Catherine J Dickinson, Julia Brown, Jane Nixon, Colin Everett, Sven Plein

**Affiliations:** 1 Department of Cardiology, Multidisciplinary Cardiovascular Research Centre & Leeds Institute of Genetics, Health and Therapeutics, Leeds, UK; 2 Division of Medical Physics, University of Leeds, Leeds, UK; 3 Department of Nuclear Cardiology, Leeds Teaching Hospitals NHS Trust, Leeds, UK; 4 Clinical Trials Research Unit, University of Leeds, Leeds, UK

## Background

Left main stem (LMS) disease occurs in approximately 5% of patients with stable angina. It confers adverse prognosis, with potential for prognostic gain with revascularization. Single-photon emission computed tomography (SPECT) and CMR fail to detect ischemia in 41% and 18% of patients with significant LMS stenosis respectively [1], likely in part because of balanced reduction in coronary perfusion. It is not known whether quantitative assessment of myocardial blood flow (MBF) can improve diagnostic rates. The CE-MARC study prospectively enrolled 752 patients with suspected coronary artery disease, scheduled to undergo CMR, SPECT and X-ray coronary angiography [2]. We assessed the diagnostic performance of visual and quantitative perfusion CMR in CE-MARC patients with LMS disease.

## Methods

All patients from the CE-MARC population with LMS disease ≥50%, or LMS equivalent disease (proximal LAD and proximal LCx ≥70%) on quantitative angiography were studied. A control group (matched for age and gender, excluding LMS or 3-vessel disease) was randomly selected from the CE-MARC population. Visual SPECT and CMR analyses were from the original, blinded read of CE-MARC. Only perfusion components of the CE-MARC CMR and SPECT protocols were analyzed. MBF was calculated offline (PMI v0.4) using the Fermi model from CMR stress perfusion images, with arterial input defined in LV blood pool, and LAD and LCx segments in the mid-LV short axis myocardial slice as tissue response.

## Results

**Figure 1:**
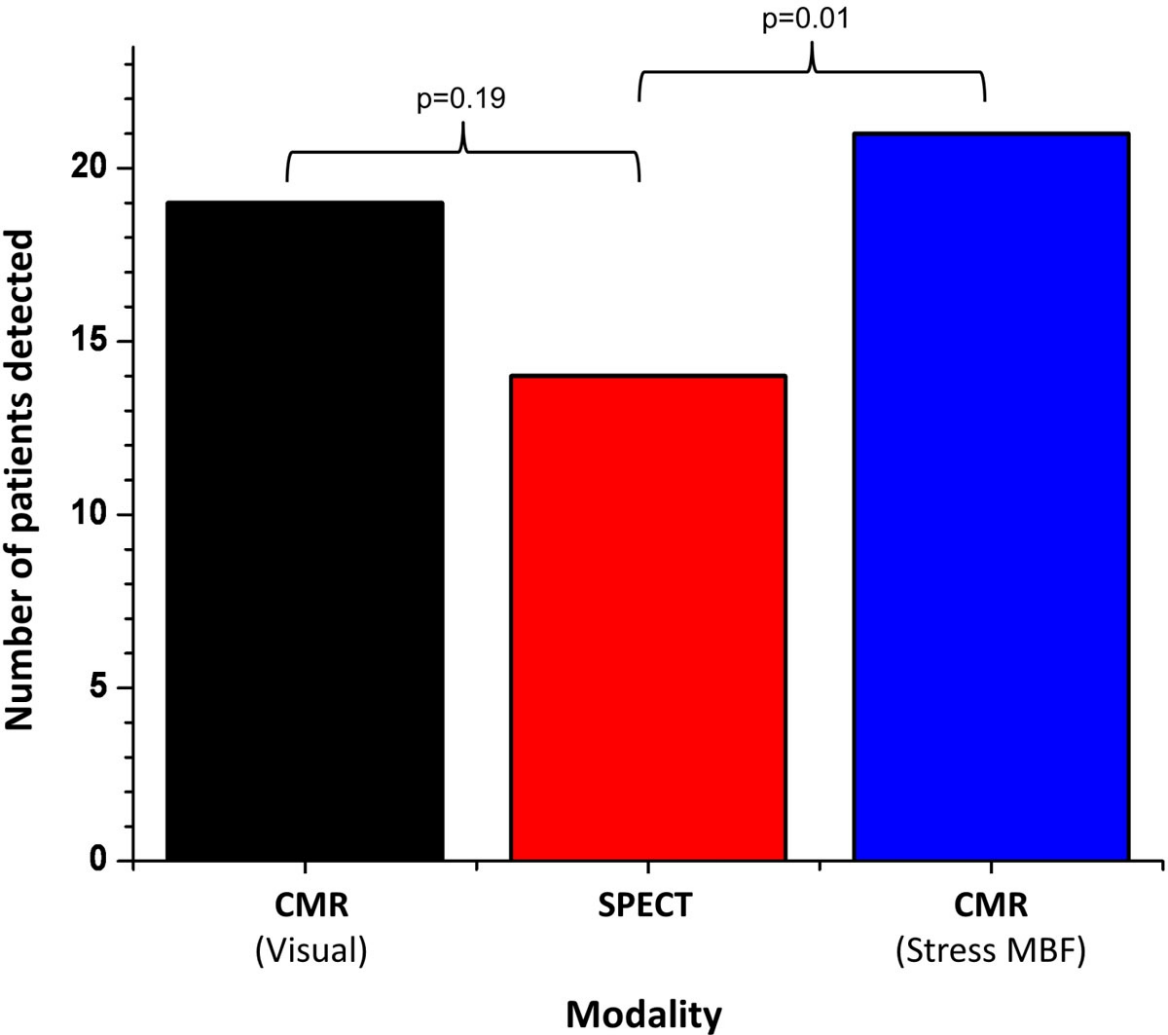
Detection rates of ischemic heart disease for CMR visual analysis, CMR MBF quantitation, and SPECT in 23 patients with LMS or LMS equivalent disease.

47 patients were included in the analysis (22 LMS, 1 LMS equivalent, 24 controls); 1 LMS patient did not have CMR. Visual detection rates for ischemia in LMS disease were non-significantly higher for CMR than SPECT (83% vs. 61%, p=0.19). On quantitative CMR perfusion analysis, stress MBF was significantly lower in LMS patients than controls (2.67±0.94 ml/g/min vs. 3.97±1.25 ml/g/min, p<0.01). ROC AUC for MBF was 0.81. An MBF cut-off of 3.78 ml/g/min was derived by Youden's Index (to objectively find optimal sensitivity and specificity). This cut-off point had sensitivity 0.96, specificity 0.67 and accuracy 0.81 for LMS disease over controls. Using this cut-off, stress MBF had higher sensitivity in LMS disease than visual SPECT analysis (96% vs. 61%, p=0.01, Figure 1), but was not significantly superior to visual CMR analysis (p=0.35).

## Conclusions

Quantitative CMR identifies LMS disease with higher sensitivity than visual SPECT analysis. Quantitative CMR analysis of MBF compared to visual CMR analysis did increase diagnostic sensitivity numerically; however this did not reach statistical significance in this small population from CE-MARC.

## Funding

CE-MARC was funded by the British Heart Foundation (BHF). JPG and SP receive an educational research grant from Philips Healthcare. SP is funded by a BHF fellowship (FS/1062/28409).

## References

[B1] Greenwood JP JCMR 2012 14 O93

[B2] Greenwood JP CE-MARC: a prospective trial Lancet 2012 379 453 460 10.1016/S0140-6736(11)61335-4 22196944 PMC3273722

